# The impact of chronic diseases and lifestyle on sarcopenia risk in older adults: a population-based longitudinal study

**DOI:** 10.3389/fmed.2025.1500915

**Published:** 2025-02-26

**Authors:** Wu-xiao Wei, Zhen-fang Mao, Meng-li Chen, Lian Meng

**Affiliations:** The First Affiliated Hospital of Guangxi University of Science and Technology, Liuzhou, China

**Keywords:** sarcopenia, chronic diseases, lifestyle, CHARLS, national longitudinal study

## Abstract

**Background:**

Sarcopenia, characterized by the gradual decline of muscle mass and strength, seriously affects the health and mobility of older adults. The purpose of this study is to investigate the risk factors for sarcopenia, particularly the relationship between chronic diseases and lifestyle factors in individuals aged 60 and over.

**Methods:**

This study used data from the Longitudinal Study on Health and Retirement in China (CHARLS) collected in 2011 and 2015. All eligible participants were classified according to the standards established by the Asian Sarcopenia Working Group in 2019. The evaluation of sarcopenia was based on a comprehensive score across five dimensions: strength, assistance in walking, rise from a chair, climb stairs, and falls. A multivariate logistic regression model was employed to explore the risk factors for sarcopenia.

**Results:**

The risk of sarcopenia is significantly influenced by multiple factors. Key findings include the association between past drinking and an increased risk of sarcopenia (HR = 2.198, 95% CI: 1.072–4.560, *p* < 0.05), indicating that individuals with a history of drinking have more than twice the risk of sarcopenia compared to non-drinkers. Chronic diseases such as stroke were also associated with a significantly elevated risk (HR = 3.137, 95% CI: 1.128–8.721, *p* < 0.05). Conversely, participation in social activities significantly reduced the risk of sarcopenia (HR = 0.482, 95% CI: 0.265–0.876, *p* < 0.05). A three-piece spline regression model revealed a nonlinear relationship between physical activity and the risk of sarcopenia, characterized by an initial decline in risk followed by an increase as physical activity levels rose. Moderate-intensity physical activity reduced the risk of sarcopenia by approximately 35% (HR ≈ 0.65). However, high-intensity physical activity led to a rebound in risk, increasing the likelihood of sarcopenia relative to moderate activity. Similarly, adequate sleep duration was associated with a reduced risk of sarcopenia, whereas excessive sleep counteracted this benefit.

**Conclusion:**

The findings underscore the critical role of lifestyle modifications and balanced physical activity in mitigating the risk of sarcopenia among older adults. Implementing targeted interventions for high-risk groups is essential to reduce the incidence of sarcopenia.

## 1 Introduction

Sarcopenia is a progressive and pervasive skeletal muscle disease, which is associated with an increased risk of physical disability, decreased quality of life, and mortality in older adults (1). It is marked by declines in muscle mass, strength, and physical function, with its prevalence increasing significantly with age (2). This condition has become a major public health problem due to its direct impact on the functional independence of older adults (3).

Recently, various risk factors for sarcopenia have been identified, including age, gender, lifestyle, and the presence of comorbidities (4–6). Demographic and social factors, such as age, gender, education level, lifestyle, and health status, have also been found to affect the risk of sarcopenia (7–10). Habits such as smoking, drinking, and participation in social activities, for example, have been shown to play a role in altering muscle strength and physical function in older adults (11, 12). The risk is further exacerbated by chronic diseases, including heart and Pulmonary diseases (13, 14). In China, where the population is rapidly aging, sarcopenia has emerged as a major health issue (15, 16).

In recent years, significant progress has been made in sarcopenia research, particularly in regions such as Europe. These studies have explored the prevalence trends, risk factors, and intervention strategies for sarcopenia, guided by standardized diagnostic criteria (1). However, their findings are challenging to directly apply to China, a country characterized by a vast population, rapid aging, and notable differences in socioeconomic structures, lifestyles, nutritional patterns, and chronic disease burdens. The unique context of the Chinese older adult population profoundly influences sarcopenia risk and mechanisms. For instance, older adults in rural areas may face higher risks of sarcopenia due to lower economic levels and insufficient nutritional intake, while urban older adult individuals may be more vulnerable to sedentary behavior and social isolation (17–19). Moreover, the high prevalence of chronic diseases such as diabetes, cardiovascular diseases, and liver diseases in China may involve complex interactions with sarcopenia, which remain underexplored in current research (20–25).

To address these gaps, this study utilizes data from the China Health and Retirement Longitudinal Study (CHARLS) to systematically examine the prevalence of sarcopenia and its major influencing factors among the Chinese older adult population. CHARLS is a nationally representative longitudinal survey that includes a diverse sample of urban and rural residents across China, with detailed data on socioeconomic status, chronic disease profiles, physical activity, and social participation. By leveraging these data, this study investigates how chronic diseases, lifestyle factors, and social engagement influence sarcopenia risk. The findings aim to provide a robust scientific foundation for developing prevention and intervention strategies tailored to the specific socioeconomic and demographic context of China, thereby addressing the global knowledge gaps on sarcopenia in Chinese populations.

## 2 Methods

### 2.1 Study design and data source

The purpose of this study is to investigate the relationship between chronic diseases, lifestyle factors, and the risk of sarcopenia in the older adult population. To ensure data quality and the reliability of the research results, data from the CHARLS were used. The CHARLS project is designed to collect high-quality microdata from Chinese households and individuals aged 45 and above, with the aim of exploring issues related to population aging in China and promoting interdisciplinary research (16). The national baseline survey of CHARLS was launched in 2011, based on a multi-stage probability sampling method using proportional probability (PPS) sampling. The sample included 450 villages, 150 counties, and 28 provinces, involving more than 17,000 individuals and approximately 10,000 families. Thereafter, CHARLS conducted follow-up surveys every 2 to 3 years. To date, four rounds of national baseline survey data have been released, including the baseline survey in 2011 (the first wave), the first follow-up survey in 2013 (the second wave), the second follow-up survey in 2015 (the third wave), and the third follow-up survey in 2018 (the fourth wave). In this study, respondents aged 60 and above were selected to investigate the relationship between sarcopenia and cognitive function in the older adult. The CHARLS project was approved by the Peking University Biomedical Ethics Review Committee, and all participants were required to sign informed consent forms. Relevant data can be accessed through the website.^1^

**Figure 1:**
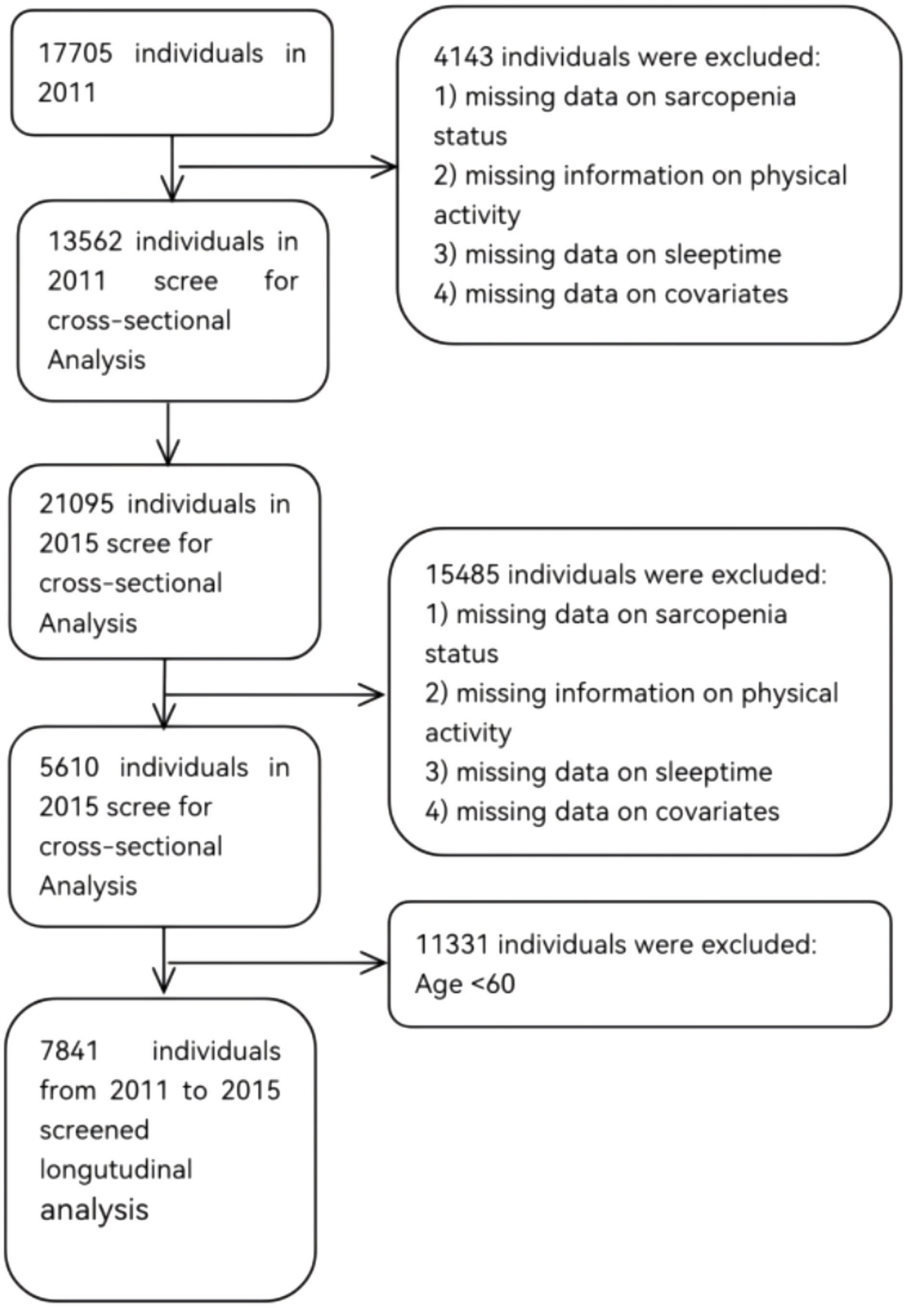
Flowchart of the sample selection process.

This study utilized data from the 2011 and 2015 waves of the CHARLS. The inclusion criteria were as follows: (1) individuals aged 60 years or older in the 2011 and 2015 datasets; and (2) availability of data necessary for sarcopenia assessment. Exclusion criteria included: (1) missing data on sarcopenia status; (2) missing information on physical activity; (3) missing data on sleeptime; (4) age below 60 years; and (5) missing data on covariates. The 2011 dataset comprised 13,562 respondents, and the 2015 dataset included 5,610 respondents. After excluding individuals younger than 60 years and those with missing data, a final sample of 7,841 participants was retained for analysis. Figure 1 illustrates the detailed sample selection process.

### 2.2 Measurement of sarcopenia

The assessment of sarcopenia was based on a comprehensive scoring system covering five dimensions: strength, assistance in walking, rise from a chair, climb stairs, and falls. Strength (2 points): Difficulty in lifting/carrying approximately 4.5 kg. 0 points = no difficulty, 1 point = some difficulty (attempted but unable to complete the task), 2 points = great difficulty, unable to complete (unable to measure due to health reasons). Assistance in walking (2 points): Difficulty in walking across a room. 0 points = no difficulty, 1 point = some difficulty (uses a walking aid or cane), 2 points = great difficulty, requires assistance, unable to complete (uses a manual or electric wheelchair). Rise from a chair (2 points): Difficulty in standing up from a bed or chair. 0 points = no difficulty, 1 point = some difficulty (unable to stand up five times), 2 points = great difficulty, unable to complete without assistance (unable to stand up). Climb stairs (2 points): Difficulty in climbing 10 steps. 0 points = no difficulty, 1 point = some difficulty, 2 points = great difficulty, unable to complete. Falls (2 points): Number of falls in the past year. 0 points = 0 falls, 1 point = 1–3 falls, 2 points = 4 or more falls. Each dimension was scored from 0 to 2, with a total score ranging from 0 to 10. According to the SARC-F scale, individuals with a total score ≥ 4 were defined as sarcopenia cases (1, 26).

### 2.3 Potential covariates

We considered socio-demographic characteristics and health-related factors based on existing knowledge. Socio-demographic characteristics included age, family size, family expenditure, family income, gender, education level (primary school and below, junior high school, senior high school and above), marital status (married, divorced, widowed, separated, and unmarried), region (eastern region, Central region, and western region), residence area (rural and urban), and medical insurance. The health-related factors included smoking, drinking, health status, social activities, dyslipidemia, and chronic diseases (Pulmonary disease, liver disease, heart disease, stroke, kidney disease, arthritis, and asthma).

### 2.4 Measurement of physical activity

Participants were asked to recall and report the time spent on various types of physical activities (METs) during the past week. Based on the standards of the International Physical Activity Questionnaire-Short Form (IPAQ-SF), daily physical activity duration of the respondents was divided into five levels: 0 min, 10–29 min, 30–119 min, 120–239 min, and ≥ 240 min, and the median of each category was used for calculation. Second, physical activity types were classified into high-intensity (e.g., hiking, running, farming), moderate-intensity (e.g., brisk walking, tai chi), and low-intensity (e.g., casual walking). The assigned metabolic equivalent values for high, moderate, and low intensity were 8.0, 4.0, and 3.3, respectively. The METs score was calculated as the product of the intensity coefficient, the number of days, and the duration (in minutes) of each physical activity.

### 2.5 Measurement of sleeptime

Sleeptime was defined based on the response to the CHARLS: “On average, how many hours of actual sleep do you get at night during the past month?” The data collected from this question were used to determine the participants’ self-reported average nightly sleeptime.

### 2.6 Statistical analysis

First of all, descriptive statistical analysis was carried out to check the basic characteristics of the samples, and sarcopenia cases were compared with non-sarcopenia cases in terms of age, gender, education level, marital status, residence, family income, smoking and drinking habits, chronic diseases, and other variables. Then, multivariate regression analysis was used to evaluate the relationship between different factors and the risk of sarcopenia. The results were expressed as hazard ratios (HR) with 95% confidence intervals (CI), showing the relative risk of sarcopenia associated with various factors. Furthermore, the dose–response relationship between sarcopenia and physical activity or sleep duration was analyzed using a cubic spline model, revealing the nonlinear relationship between these behavioral factors and the risk of sarcopenia. All statistical analyses were conducted using stata 16.0, with the significance level set at 0.05.

## 3 Results

### 3.1 Basic characteristics analysis

From the analysis in Tables 1, 2, significant differences in several key variables were observed between sarcopenia cases and non-sarcopenia cases. In terms of age, the average age of sarcopenia cases was 72.66 years, significantly higher than the 67.81 years for non-sarcopenia cases (*p* < 0.001). Regarding gender, the proportion of women in the sarcopenia group was higher (67.57%) than in the non-sarcopenia group (48.54%) (*p* < 0.001). Compared to women, the risk of sarcopenia in men was significantly lower (HR = 0.597, 95% CI: 0.336–1.061). In terms of education level, 87.26% of sarcopenia cases had a primary school education and below, significantly higher than the 79.06% in non-sarcopenia cases (*p* = 0.005). With regard to lifestyle, 65.64% of sarcopenia patients had never smoking, compared with 55.88% of non-sarcopenia patients (*p* < 0.001). The risk of sarcopenia was significantly increased among past drinking (HR = 2.198, 95% CI: 1.072–4.560), while no significant change in risk was observed among current drinking (HR = 1.160, 95% CI: 0.605–2.223). The results were consistent with the harmful effects of excessive drinking and the potential benefits of moderate drinking. Regarding health status, 70.66% of sarcopenia cases reported poor health, much higher than the 32.27% of non-sarcopenia cases (*p* < 0.001). Additionally, the proportions of individuals with Pulmonary disease, heart disease, and arthritis were significantly higher in the sarcopenia group than in the non-sarcopenia group, at 21.62% (*p* < 0.001), 21.62% (*p* = 0.004), and 48.65% (*p* < 0.001), respectively. In terms of social activities, 71.81% of sarcopenia cases did not participate in social activities, significantly higher than the 49.75% in non-sarcopenia cases (*p* < 0.001).

**Table 1:** Analysis of basic characteristics.

Category	Subcategory	Total (*n* = 7,841)	Non-sarcopenia (*n* = 7,582)	Sarcopenia (*n* = 259)	*p*-value
Age (years, M ± SD)	–	67.970 ± 6.602	67.809 ± 6.496	72.660 ± 7.861	<0.001
Family size M ± SD	–	3.084 ± 1.821	3.080 ± 1.818	3.201 ± 1.910	0.292
Family expenditure M ± SD	–	9.432 ± 1.387	9.439 ± 1.382	9.219 ± 1.502	0.012
Family income M ± SD	–	7.700 ± 3.353	7.719 ± 3.349	7.133 ± 3.433	0.006
Gender	Female	3,855 (49.16)	3,680 (48.54)	175 (67.57)	<0.001
	Male	3,986 (50.84)	3,902 (51.46)	84 (32.43)	
Education level	Primary school and below	6,220 (79.33)	5,994 (79.06)	226 (87.26)	0.005
	Junior high school	1,027 (13.10)	1,008 (13.29)	19 (7.34)	
	Senior high school and above	594 (7.58)	580 (7.65)	14 (5.41)	
Marital status	Divorced/widowed/separated/unmarried	1,603 (20.44)	1,521 (20.06)	82 (31.66)	<0.001
	Married	6,238 (79.56)	6,061 (79.94)	177 (68.34)	
Region	Eastern region	2,623 (33.45)	2,545 (33.57)	78 (30.12)	0.378
	Central region	2,883 (36.77)	2,788 (36.77)	95 (36.68)	
	Western region	2,335 (29.78)	2,249 (29.66)	86 (33.20)	
Residence area	Rural	4,713 (60.11)	4,538 (59.85)	175 (67.57)	0.013
	Urban	3,128 (39.89)	3,044 (40.15)	84 (32.43)	
Medical insurance	No	640 (8.16)	617 (8.14)	23 (8.88)	0.668
	Yes	7,201 (91.84)	6,965 (91.86)	236 (91.12)	
Smoking	Never	4,407 (56.20)	4,237 (55.88)	170 (65.64)	<0.001
	Past smoking	1,033 (13.17)	990 (13.06)	43 (16.60)	
	Current smoking	2,401 (30.62)	2,355 (31.06)	46 (17.76)	
Drinking	Never	4,432 (56.52)	4,267 (56.28)	165 (63.71)	<0.001
	Past drinking	966 (12.32)	921 (12.15)	45 (17.37)	
	Current drinking	2,443 (31.16)	2,394 (31.57)	49 (18.92)	
Health status	Poor	2,630 (33.54)	2,447 (32.27)	183 (70.66)	<0.001
	Fair	3,684 (46.98)	3,618 (47.72)	66 (25.48)	
	Good	1,032 (13.16)	1,027 (13.55)	5 (25.48)	
	Very good	475 (6.06)	470 (6.20)	5 (25.48)	
	Excellent	20 (0.26)	20 (0.26)	0 (0.00)	
Social activities	Not participated	3,958 (50.48)	3,772 (49.75)	186 (71.81)	<0.001
	Participated	3,883 (49.52)	3,810 (50.25)	73 (28.19)	
Dyslipidemia	No	7,039 (89.77)	6,814 (89.87)	225 (86.87)	0.117
	Yes	802 (10.23)	768 (10.13)	34 (13.13)	
Pulmonary disease	No	6,796 (86.67)	6,593 (86.96)	203 (78.38)	<0.001
	Yes	1,045 (13.33)	989 (13.04)	56 (21.62)	
Liver disease	No	7,517 (95.87)	7,272 (95.91)	245 (94.59)	0.295
	Yes	324 (4.13)	310 (4.09)	14 (5.41)	
Heart disease	No	6,638 (84.66)	6,435 (84.87)	203 (78.38)	0.004
	Yes	1,203 (15.34)	1,147 (15.13)	56 (21.62)	
Stroke	No	7,593 (96.84)	7,371 (97.22)	222 (85.71)	<0.001
	Yes	248 (3.16)	211 (2.78)	37 (14.29)	
Kidney disease	No	7,328 (93.46)	7,085 (93.45)	243 (93.82)	0.809
	Yes	513 (6.54)	497 (6.55)	16 (6.18)	
Arthritis	No	5,005 (63.83)	4,872 (64.26)	133 (51.35)	<0.001
	Yes	2,836 (36.17)	2,710 (35.74)	126 (48.65)	
Asthma	No	7,455 (95.08)	7,218 (95.20)	237 (91.51)	0.007
	Yes	386 (4.92)	364 (4.80)	22 (8.49)	

**Table 2:** Risk of dermatomyositis incidence by different classifications.

Category	Subcategory	HR	95%CI
Gender	Female	1	Reference
	Male	0.597	0.336–1.061
Education level	Primary school and below	1	Reference
	Junior high school	1.286	0.599–2.762
	Senior high school and above	1.118	0.398–3.136
Marital status	Divorced/widowed/separated/unmarried	1	Reference
	Married	0.570	0.310–1.046
Region	Eastern region	1	Reference
	Central region	0.724	0.368–1.424
	Western region	0.894	0.454–1.759
Residence area	Rural	1	Reference
	Urban	0.726	0.400–1.320
Medical insurance	No	1	Reference
	Yes	0.535	0.240–1.191
Smoking	Never	1	Reference
	Past smoking	1.470	0.717–3.017
	Current smoking	0.621	0.303–1.275
Drinking	Never	1	Reference
	Past drinking	2.198	1.072–4.560
	Current drinking	1.160	0.605–2.223
Social activities	Not participated	1	Reference
	Participated	0.482	0.265–0.876
Dyslipidemia	No	1	Reference
	Yes	1.011	0.401–2.551
Pulmonary disease	No	1	Reference
	Yes	1.499	0.727–3.088
Liver disease	No	1	Reference
	Yes	2.631	1.043–6.635
Heart disease	No	1	Reference
	Yes	1.812	0.945–3.476
Stroke	No	1	Reference
	Yes	3.137	1.128–8.721
Arthritis	No	1	Reference
	Yes	1.722	0.983–3.014
Asthma	No	1	Reference
	Yes	1.286	0.400–4.135

As shown in Supplementary Table 1, to assess potential multicollinearity among the explanatory and control variables, variance inflation factor (VIF) values were calculated. Supplementary Table 1 presents the VIF values for all variables. The maximum and average VIF values were 1.91 and 1.18, respectively, both of which are well below the commonly accepted threshold of 10. Therefore, it can be concluded that multicollinearity does not pose a concern in this study.

To address potential sample selection bias in this study, propensity score matching (PSM) was applied. Samples with chronic diseases were designated as the treatment group, while those without chronic diseases were assigned to the control group. Nearest-neighbor matching was performed to pair the treatment and control groups. As shown in Supplementary Table 2, the covariate balance tests before and after matching indicate that the absolute values of most covariate t-statistics significantly decreased post-matching, reflecting minimal differences in covariates between the two groups. Additionally, the post-matching bias rates further confirm that the differences between the treatment and control groups were substantially reduced.

### 3.2 Dose–response relationship (cubic spline model)

#### 3.2.1 Sarcopenia and physical activity

**Figure 2:**
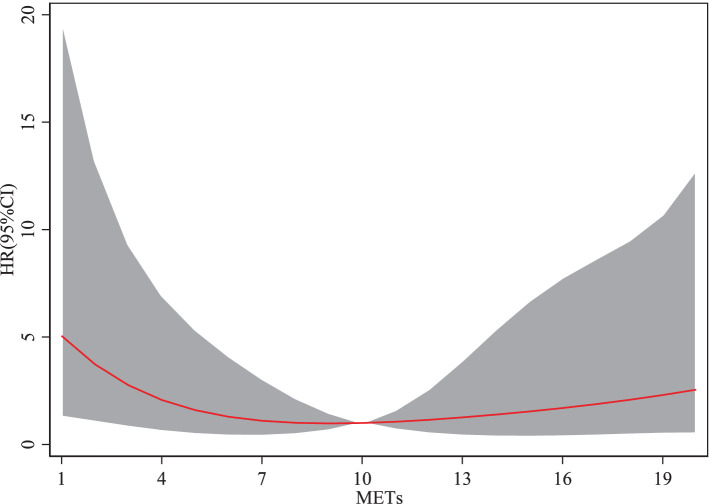
Trivariate spline model of the association between physical activity and dermatomyositis.

From the Figure 2, the cubic spline model illustrating the association between physical activity and the risk of sarcopenia reveals a non-linear relationship, rather than a monotonic increase or decrease. When the metabolic equivalent of task ranges from 1 to 10, an increase in METs is associated with a decreasing risk of sarcopenia. However, when METs exceed 10, the risk of sarcopenia begins to increase with further increases in METs. The HR for sarcopenia demonstrates a trend of initially decreasing and then increasing as METs rise. Moderate-intensity physical activity reduced the risk of sarcopenia by approximately 35% (HR ≈ 0.65). However, high-intensity physical activity led to a rebound in risk, increasing the likelihood of sarcopenia relative to moderate activity. This finding suggests that engaging in moderate physical activity helps reduce the risk of sarcopenia among older adults, whereas excessive physical activity may elevate the risk.

#### 3.2.2 Sarcopenia and sleeptime

**Figure 3:**
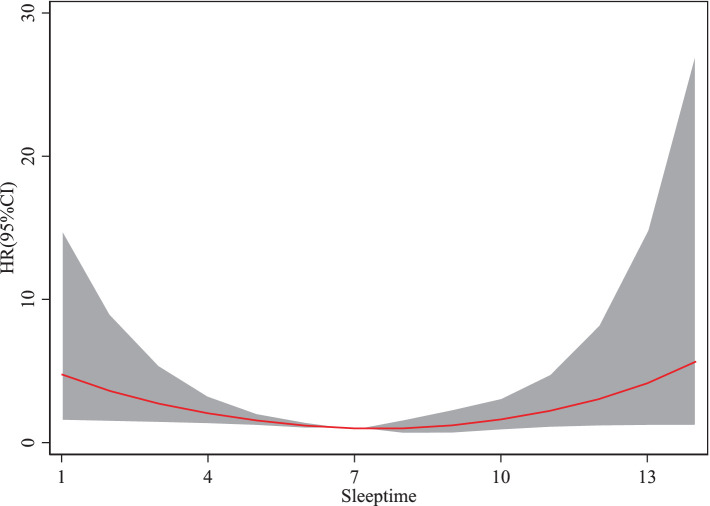
Trivariate spline model of the association between sleeptime and dermatomyositis.

As shown in Figure 3, sleep durations between 1 and 7 h are associated with a decreasing risk of sarcopenia as duration increases. However, when sleep duration exceeds 7 h, the risk of sarcopenia begins to rise with further increases. The HR for sarcopenia exhibits a trend of initially decreasing and subsequently increasing with longer sleep durations. These findings suggest that adequate sleep duration benefits older adults by reducing the risk of sarcopenia, whereas excessive sleep duration may increase the risk.

## 4 Discussion

With increasing age, the incidence of sarcopenia shows a significant upward trend worldwide. There are considerable differences in the prevalence of sarcopenia across different countries and regions. According to European research data, the prevalence of sarcopenia is generally higher, which is related to the earlier aging of society in these regions. The prevalence of sarcopenia in individuals over 65 years old ranges from about 11 to 50%, and this variation is closely related to the study population, diagnostic criteria, and lifestyle habits. In North America, it is reported that the incidence of sarcopenia in similar age groups ranges from 13 to 24% (27). However, due to the rapid aging of the population, sarcopenia is a more serious problem in Asian countries. The prevalence of sarcopenia among older adults over 65 years old in Japan is 18.1% (6, 28). One study in South Korea showed that the prevalence rates among men and women were 15.4 and 22.1%, respectively (29). Research based on data from the CHARLS shows that the prevalence of sarcopenia among older adults aged 60 and over in China is 3.30%. The data indicate that the prevalence of sarcopenia in China is relatively low compared with Western countries. However, because of China’s large population, the actual number of sarcopenia patients may far exceed that of other countries. This difference may be related to factors such as traditional Chinese diets, levels of physical activity, and healthy lifestyle habits (5).

As people age, muscle mass and strength gradually decline, which is one of the core characteristics of sarcopenia. Several studies show that muscle mass decreases by about 8% every ten years after the age of 40, and this rate accelerates to about 15% every ten years after the age of 60 (5). The underlying causes of this process include the decline in neuromuscular function, decreased muscle protein synthesis, and the accumulation of chronic inflammation (30). Among older adults, declining hormone levels, such as growth hormone and testosterone, are also considered major factors that accelerate muscle loss (31).

Chronic diseases are key factors that accelerate muscle loss, especially in patients with heart disease and chronic obstructive pulmonary disease (COPD). Chronic inflammatory states and metabolic disorders typically promote muscle tissue catabolism and further aggravate the onset of sarcopenia (13, 32, 33). In addition, individuals with liver disease and stroke have a significantly higher risk of sarcopenia than healthy individuals. The liver plays a key role in protein metabolism, detoxification, and energy storage, which makes liver disease closely related to sarcopenia. Liver disease leads to malnutrition, decreased protein synthesis, and chronic inflammation, all of which can contribute to sarcopenia (34–37). Nishikawa et al. conducted a study in Japan, demonstrating that systemic inflammation and impaired protein metabolism accelerate muscle wasting, leading to a high prevalence of sarcopenia in patients with liver disease (33). Similarly, Fülster et al. reported a significant prevalence of sarcopenia in patients with chronic heart failure, primarily driven by reduced physical activity and increased systemic catabolism (38). These findings align with our study, further confirming that chronic diseases markedly increase the risk of developing sarcopenia.

Sarcopenia and chronic diseases have a bidirectional relationship, mutually influencing each other (39). Yu et al. demonstrated that sarcopenia significantly increases the risk of disease progression in patients with chronic liver disease, leading to worse clinical outcomes (23, 40). Furthermore, Sepúlveda-Loyola et al. reported that sarcopenia is strongly associated with a higher risk of chronic obstructive pulmonary disease (COPD), exacerbating patients’ overall health status (32). Similarly, Damluji et al. highlighted that sarcopenia is closely linked to accelerated progression of cardiovascular diseases, increased mortality, higher fall incidence, and reduced quality of life, particularly in older adults (13). Collectively, these studies underscore the pivotal role of sarcopenia in driving the progression of chronic diseases.

Lifestyle factors play an important role in the pathogenesis and progression of sarcopenia. Proper physical activity helps maintain muscle mass, while both sedentary behavior and excessive activity increase the risk of sarcopenia. Studies have shown that moderate-intensity exercise, such as brisk walking or strength training, helps reduce the risk of sarcopenia, while excessive high-intensity exercise may lead to muscle injury, thereby increasing the risk (1, 5, 7, 41, 42). Additionally, the quality and duration of sleep are closely related to the occurrence of sarcopenia. Both insufficient and excessive sleep affect the endocrine system and inflammatory responses, accelerating muscle loss (9, 43–45). Furthermore, this study found that older adults who do not participate in social activities have a significantly higher risk of sarcopenia. Social isolation may exacerbate mental health problems, such as depression and anxiety, and further impact the physical activity levels and overall health of older adults (46, 47).

Although this study provides valuable insights into the prevalence and influencing factors of sarcopenia among older adults in China, it has some limitations. First, this study is based on samples of older adults primarily from specific areas, which may not fully reflect the situation of all older adults in the country, particularly those in remote or economically underdeveloped regions. Second, some lifestyle data (e.g., physical activity, smoking, and drinking) relied on self-reports from respondents, which carry the risk of recall bias and inaccurate reporting. This may lead to misclassification of some variables and affect the accuracy of the research. Third, some analyses are cross-sectional, making it difficult to determine causal relationships. The relationship among chronic diseases, lifestyle factors, and sarcopenia may be bidirectional, and this interaction cannot be fully captured by a cross-sectional design. Although this study adjusted for several potential confounders (e.g., age, gender, chronic diseases), other uncontrolled factors (such as genetic background and dietary patterns) may still influence the occurrence of sarcopenia. Moreover, this study used the SARC-F scale to evaluate sarcopenia. While this screening tool is highly practical, its sensitivity and specificity may be insufficient, potentially missing early or mild cases of sarcopenia.

## 5 Conclusion

This study identified the main risk factors for sarcopenia in older adults, including age, chronic diseases (such as lung and heart diseases), lifestyle factors (such as physical activity and sleep), and social participation. The results suggest that preventing sarcopenia should focus on lifestyle adjustments, particularly moderate exercise and the management of chronic diseases. Large-scale longitudinal research is needed in the future to explore the long-term impact of various risk factors and to formulate personalized intervention measures aimed at improving the quality of life for older adults and reducing the health risks associated with sarcopenia.

## Data Availability

The datasets presented in this study can be found in online repositories. The names of the repository/repositories and accession number(s) can be found at: https://charls.pku.edu.cn/en.

## Footnotes

^1^ http://charls.pku.edu.cn/index.htm
