# Supplementary material for: The impact of chronic diseases and lifestyle on sarcopenia risk in older adults: a population-based longitudinal study
Source: Front Med (Lausanne). 2025 Feb 26;12:1500915. doi: 10.3389/fmed.2025.1500915 (PMC11897525; doi:10.3389/fmed.2025.1500915)
Supplement: Supplementary file 1 [file Data_Sheet_1.pdf]

Supplementary Appendix

This appendix has been provided by authors to give readers additional information about their work.

Supplement to: The impact of chronic diseases and lifestyle on sarcopenia risk in older adults: a population-based longitudinal study

Supplementary Table 1 Multicollinearity Test

Variable	VIF	1/VIF
Gender	1.91	0.524094
Smoking	1.64	0.611371
Drinking	1.34	0.746227
Education level	1.23	0.814786
Family expenditure	1.20	0.830633
Family size	1.20	0.834425
Residence area	1.18	0.84807

Pulmonary Disease	1.18	0.848414
Marital status	1.18	0.850395
Age	1.16	0.862136
Health status	1.16	0.864596
Family income	1.15	0.868882
Asthma	1.14	0.874075
Arthritis	1.11	0.897418
Heart Disease	1.11	0.901233
Dyslipidemia	1.09	0.919388
Region	1.09	0.921433
Kidney Disease	1.06	0.947463
Sleeptime	1.05	0.950705
Social activities	1.05	0.952994
METs	1.05	0.95583
Liver Disease	1.03	0.968286
Stroke	1.03	0.974307
Medical insurance	1.02	0.976718
Mean VIF	1.18	

Supplementary Table 2 Balance Test

Variable	Matched	Treated	Control	%bias	bias	t	p>t
Age	U	67.969	67.97	0	16406.7	-0.01	0.994
	M	67.973	68.154	-2.7		-1.28	0.199
Gender	U	0.48917	0.53389	-9	82.5	-3.92	0
	M	0.48949	0.48167	1.6		0.74	0.459
Education level	U	1.2649	1.3059	-6.9	92.3	-3.02	0.003
	M	1.2649	1.268	-0.5		-0.26	0.797
Marital status	U	0.79205	0.80024	-2	15.4	-0.89	0.374
	M	0.79213	0.7852	1.7		0.8	0.422
Region	U	2.0241	1.8823	17.9	89.8	7.86	0
	M	2.0235	2.0379	-1.8		-0.86	0.388
Residence area	U	0.39602	0.40279	-1.4	72.4	-0.61	0.545
	M	0.39584	0.39771	-0.4		-0.18	0.856
Medical insurance	U	0.92741	0.90636	7.6	99.3	3.37	0.001
	M	0.92736	0.9275	-0.1		-0.03	0.979
Smoking	U	1.7228	1.7726	-5.5	87.7	-2.44	0.015
	M	1.7233	1.7172	0.7		0.32	0.745
Drinking	U	1.7179	1.7842	-7.3	68	-3.23	0.001
	M	1.7181	1.697	2.3		1.13	0.259
Health status	U	1.7233	2.1935	-56.7	99.1	-25.13	0
	M	1.7237	1.7278	-0.5		-0.25	0.801
Social activities	U	0.49073	0.50119	-2.1	45.6	-0.92	0.359
	M	0.49039	0.4847	1.1		0.54	0.59
Family size	U	3.1036	3.0568	2.6	67.6	1.13	0.259
	M	3.1044	3.1196	-0.8		-0.39	0.697
Family expenditure	U	9.4884	9.3574	9.3	97.6	4.14	0

	M	9.4871	9.484	0.2		0.12	0.908
Family income	U	7.812	7.5511	7.7	94.4	3.41	0.001
	M	7.8107	7.7961	0.4		0.21	0.831
